# Antifungal Susceptibility Profile of *Aspergillus* Strains Isolated From the Lower Respiratory Tract in Eastern Indian Patients: A Hospital‐Based Study

**DOI:** 10.1002/mbo3.70136

**Published:** 2025-11-18

**Authors:** Aishwarya Nikhil, Sradha Choudhury, Mohit Bhatia, Atul Kumar Tiwari, Ritika Srivastava, Abhirami Prasad, Ragini Tilak, Munesh K. Gupta, Roger J. Narayan

**Affiliations:** ^1^ Mycology research group, Department of Microbiology, Institute of Medical Sciences Banaras Hindu University Varanasi India; ^2^ Department of TB & Respiratory Diseases Sir Sunderlal Hospital (BHU) Varanasi India; ^3^ Department of Chemistry Indian Institute of Technology (BHU) Varanasi India; ^4^ Joint Department of Biomedical Engineering North Carolina State University Raleigh North Carolina USA

**Keywords:** antifungal susceptibility, aspergillosis, bronchiectasis, epidemiological cutoff value, tuberculosis

## Abstract

Respiratory aspergillosis refers to a range of infections, from allergic to chronic and invasive, which can be life‐threatening and are primarily caused by *Aspergillus fumigatus* and *Aspergillus flavus*. Other species, including *Aspergillus terreus, Aspergillus nidulans*, and *Aspergillus versicolor*, have also been implicated in respiratory infections. Treatment for chronic to invasive pulmonary aspergillosis typically involves azole antifungal drugs, although studies have shown varying minimum inhibitory concentrations (MIC) for these medications, with a growing concern over voriconazole resistance. During the period from August 2022 to May 2024, characteristic hyphae were detected in 7.2% of lower respiratory samples, with culture positivity in 12.8%, including early morning sputum and bronchoalveolar lavage fluid samples. *A. flavus* (*n* = 282) was the most frequently isolated species, followed by *A. fumigatus* (*n* = 86). Additionally, a seasonal trend was observed for *Aspergillus* infections, with peaks in April and September. The MIC of itraconazole, voriconazole, posaconazole, amphotericin B, ravuconazole, and caspofungin were assessed for the isolated *Aspergillus* species. A higher MIC of amphotericin B was observed against *A. flavus* and *A. terreus*, whereas azoles exhibited a relatively lower MIC. Caspofungin and posaconazole exhibited the lowest MIC against the isolated *Aspergillus* species. Therefore, it is crucial to identify the causative fungi and determine the antifungal MIC for *Aspergillus* species responsible for lower respiratory tract infections. This study emphasizes the significance of respiratory aspergillosis in TB‐endemic regions of Eastern India.

## Graphical abstract

Treatment for chronic to invasive pulmonary aspergillosis typically involves azole antifungal drugs, although studies have shown varying minimum inhibitory concentrations (MIC) for these medications, with a growing concern over voriconazole resistance. The MIC of itraconazole, voriconazole, posaconazole, amphotericin B, ravuconazole, and caspofungin were assessed for the isolated Aspergillus species.

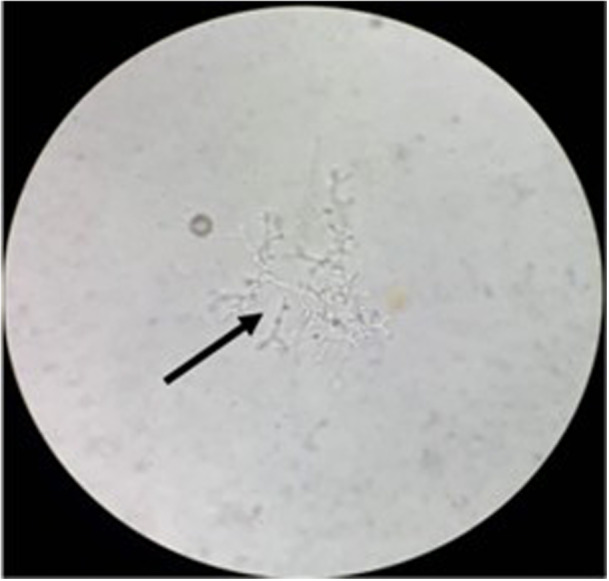

## 1 Introduction

Lower respiratory tract infections (LRTI) are a significant source of illness and death among immunocompetent and immunosuppressed individuals (Safiri et al. 2023). However, since the COVID‐19 pandemic, there has been a noticeable increase in the incidence of fungal LRTI. In developing nations such as India, *Mycobacterium tuberculosis* is the leading cause of chronic lung infections, which present as a cough with sputum and a rise in temperature during the evening. Approximately two‐thirds of suspected pulmonary tuberculosis cases receive bacteriological confirmation; however, one‐fourth are treated based on clinical suspicion alone (WHO 2023). This highlights the necessity of investigating the microbial causes of these instances. The fungus *Aspergillus* is known to cause allergic, chronic, and invasive respiratory diseases. In countries where pulmonary tuberculosis is prevalent, such as India, Denning et al. (2023) reported 363,601 new cases of chronic pulmonary aspergillosis (CPA) in 2019, resulting in 42,766 deaths that year. Additionally, they noted a 5‐year prevalence of CPA of 1,575,716 cases, with an extra 100,715 deaths annually (Denning et al. 2023). CPA frequently occurs in patients with pre‐existing cavities, bronchiectasis, and obstructive pulmonary disease. Furthermore, factors such as SARS‐CoV‐2, influenza viral infections, steroid use, diabetes, and immunosuppressive medications increase susceptibility (Jhun et al. 2017). CPA symptoms often resemble those of pulmonary tuberculosis. *Aspergillus* is an environmental saprotrophic fungus, with over 60 species reported to cause respiratory infections (Denning et al. 2023). However, *A. fumigatus, A. flavus, A. niger, A. terreus*, and *A. nidulans* are the primary causative agents of pulmonary aspergillosis (Ullmann et al. 2018; Zanganeh et al. 2018). Among these, *A. fumigatus* is the most prevalent species causing this invasive fungal infection, whereas *A. flavus* is responsible for CPA (Lamoth et al. 2017). These fungal infections are treated with triazoles, echinocandins, and polyenes, along with addressing underlying risk factors. Most cases respond well to triazoles, but increasing azole resistance in *Aspergillus* is an evolving concern. In India, 0.8% of azole‐resistant *A. fumigatus* strains have been reported to cause human infection (Dabas et al. 2018). As such, there is limited information on pulmonary aspergillosis in tuberculosis‐endemic regions of Eastern India. Consequently, we designed this prospective hospital‐based study to identify the range of *Aspergillus* species causing human respiratory infections in Eastern India and to assess the antifungal susceptibility profiles of various antifungal agents at a tertiary care center serving eastern Uttar Pradesh, western Bihar, and Jharkhand states of India.

## 2 Materials and Methods

### 2.1 Materials

All materials and antifungal drugs used in this study were of analytical grade. The antifungal drugs, amphotericin B (AMB), itraconazole (ITZ), posaconazole (PCZ), ravuconazole (RUV), caspofungin (CAS), and voriconazole (VOR) were purchased from Sigma‐Aldrich (Mumbai, India). Potato Dextrose Agar (PDA) and Roswell Park Memorial Institute 1640 medium containing l‐glutamine and without sodium bicarbonate (RPMI) with 0.165 mol/L 3‐(N‐morpholino) propane sulfonic acid buffer having 7.0 pH were purchased from HiMedia Laboratories Limited (Mumbai, India). Sterile 96‐well flat‐bottom microtiter plates and other plastic wares were procured from Tarson Product Private Limited (Kolkata, West Bengal, India).

### 2.2 Ethics Statement

The Institutional Ethical Committee (Dean/2021/EC/3003, dated October 29, 2021) approved this prospective study. All *Aspergillus* strains used in this study were isolated from patients with respiratory illnesses. This study complied with the Helsinki Declaration on patient health, dignity, privacy, and confidentiality of personal information.

### 2.3 Study Population and General Considerations

This prospective study was conducted in the Department of Microbiology and the Department of Tuberculosis and Respiratory Medicine at the Institute of Medical Sciences, Banaras Hindu University, Varanasi, India, from August 2022 to May 2024. Initially, we obtained written informed consent from patients who presented with complaints of persistent cough and sputum production lasting more than a month, with or without accompanying symptoms such as fever, breathlessness, and/or hemoptysis. We also recorded demographic details, including age, sex, and previous medical history, which encompassed immune suppression, viral infections such as SARS‐CoV‐2, influenza virus, respiratory tuberculosis or post‐TB sequelae, lung parenchymal disorders, diabetes, history of alcohol consumption, smoking, allergies, medications such as steroids and other immunosuppressive agents, and any prior hospitalizations. All patients suspected of having a fungal infection underwent radiological and microbiological evaluations. For radiological assessment, High‐Resolution Computed Tomography of the thorax was performed to identify cavities, nodules, infiltrations, wedge‐shaped infarcts, air crescent signs, halo or reverse halo signs, aspergilloma, or consolidation. For microbiological evaluation, bronchoalveolar lavage (BAL) and early morning sputum samples were processed according to standard mycological procedures (ICMR 2019). Briefly, respiratory samples were examined for characteristic hyphae using potassium hydroxide (KOH) and calcofluor white wet mounts. Additionally, the respiratory samples were inoculated on blood agar and SDA medium and incubated at 28°C in a biological oxygen demand incubator. Once fungal growth was observed on the medium, the fungi were identified based on their culture characteristics and morphological features, such as the length of the conidiophores, smooth or rough walls, vesicle shape, presence or absence of metulae, arrangement of phialides, and conidia characteristics using a lactophenol cotton blue wet mount. Final identification was achieved through slide culture, and the strains were stored in 20% glycerol broth at −20°C for further analysis. Based on clinical, radiological, and microbiological evaluations, we categorized the patients into the following groups: aspergilloma, allergic bronchopulmonary aspergillosis, chronic pulmonary aspergillosis, fungal pneumonia, and proven or probable invasive pulmonary aspergillosis, following the criteria set by the European Organization for Research and Treatment of Cancer/Invasive Fungal Infections Cooperative Group and the National Institute of Allergy and Infectious Diseases Mycoses Study Group.

### 2.4 *In Vitro* Antifungal Susceptibility Testing

Antifungal drug susceptibility was evaluated against the isolated fungal species following CLSIM38A_3_ guidelines with minor modifications (Clinical and Laboratory Standards Institute CLSI 2022). For standardization of the MIC protocol, we used *Candida parapsilosis* (ATCC 22019), *Candida krusei* (ATCC 6258), and *Aspergillus flavus* (ATCC 204304). Antifungal drug powders, including amphotericin B (AMB), itraconazole (ITZ), posaconazole (PCZ), ravuconazole (RUV), caspofungin (CAS), and voriconazole (VOR), were procured from Sigma Aldrich Chemicals Private Limited, Mumbai, India. Initially, the drug powders were dissolved in DMSO and then further diluted in RPMI 1640 to obtain drug concentrations in the range of 0.03–16 µg/mL, except for CAS, which was diluted to obtain a range of 0.015–8 µg/mL. For *Aspergillus* inoculum preparation, the isolated *Aspergillus* strains were grown on PDA at 35°C for 7 days. Subsequently, the conidia were harvested in a sterile normal saline solution containing 0.1% Tween 20, and the optical density of 0.09 to 0.13 was measured at 530 nm. Subsequently, the suspension was further diluted 1:50 to achieve a final count of 5 × 10^4^ conidia/mL using a hemocytometer.

The MIC of the above‐mentioned drugs was determined using the serial broth microdilution method in a sterile 96‐well flat‐bottom microtiter plate. Initially, 100 µL of each diluted drug in RPMI 1640 was suspended in each well. Then, 100 µL of the conidial suspension (5 × 10^4^ conidia/mL) was dispensed into each well, and the microtiter plate was incubated at 35°C for 48 h. The minimum drug concentration that completely inhibited fungal growth was considered the MIC for azoles and AMB. However, the minimum effective concentration (MEC) of CAS was determined microscopically. Here, the minimum drug concentration of CAS resulted in compact, small, and rounded compact hyphal growth compared to normal hyphal growth at 24 h. of incubation. Subsequently, we calculated the geometric mean, MEC_50_, MIC_50_, MEC_90,_ and MIC_90_ ranges as well as median values for all six antifungal drugs against isolated *Aspergillus* species. Moreover, we categorized the isolated *Aspergillus* strains into wild‐type (WT) and non‐wild‐type (NWT) for CAS, PCZ, VOR, ITZ, and AMB based on epidemiological cut‐off values (ECVs) in the CLSI M59 guidelines (Clinical and Laboratory Standards Institute CLSI 2018).

### 2.5 Data Analysis

ECVs for antifungal susceptibility testing were analyzed according to the CLSI M59 guidelines. The MEC_50_, MIC_50_, MEC_90, and_ MIC_90_ ranges and median values were calculated for all six antifungal drugs against *the Aspergillus* species. We also determined the number of wild‐type and NWT strains of *A. fumigatus*, *A. flavus*, *A. terreus*, and *A. versicolor* strains for CAS, PCZ, VOR, ITZ, and AMB.

### 2.6 Statistical Analysis

The data were independently cross‐checked using MS Excel (Microsoft, Redmond, Washington, USA) to minimize possible errors. Qualitative findings are presented as absolute percentages, whereas quantitative findings are expressed as median values and interquartile ranges. Additionally, the ranges of MIC/MEC, geometric mean, absolute number, and percentage of MIC/MEC_50_ and MIC/MEC_90_ values were calculated for each tested drug. Tukey's multiple comparison test and two‐way ANOVA were used in the statistical study to assess variations in the MIC_50_ and MIC_90_ values across several antifungal drugs. Statistical significance was set at *p* < 0.05 after data analysis and visualization using GraphPad Prism v.8.0.2 (GraphPad Software, San Diego, CA, USA).

## 3 Results

### 3.1 Demographic Data of the Patients

**Figure 1:**
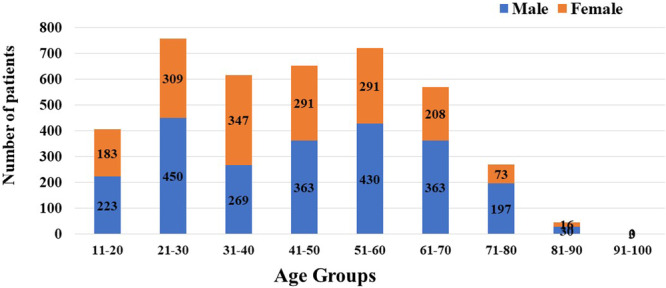
Age and gender distribution of the patients having suspected respiratory aspergillosis.

Between August 2022 and May 2024, we collected 4046 unique respiratory samples from patients suspected of having respiratory aspergillosis at the Department of TB and Respiratory Medicine, Sir Sunder Lal Hospital, Institute of Medical Sciences, Banaras Hindu University. Categorically, 2780 (68.7%) were early morning sputum samples and 1,266 (31.3%) were BAL samples. The male‐to‐female ratio among the patients was 1.35:1, and the average age was 44.8 years, with a standard deviation of 18.24 years and a standard error of 0.28 (Figure 1). All patients with pulmonary aspergillosis reported symptoms of cough, sputum production, and breathlessness, and 30% experienced hemoptysis. Table 1 outlines the predisposing risk factors for the disease. Males accounted for 57.53% of the samples, a figure associated with occupational exposure, smoking, and other risk factors. Nearly all patients reported cough, sputum production, and breathlessness, with approximately 30% experiencing hemoptysis. Among the patients, 34.3% had previously received anti‐TB treatment. Notably, 22% had a history of SARS‐CoV‐2 infection, leading to hospitalization of 79 individuals. Additional risk factors included pulmonary fibrosis and lung parenchymal disorders, neutropenia, immune suppression, malignancy, and diabetes mellitus, observed in 34%, 15%, 10%, 34.54%, and 43.03% of cases, respectively. Addiction and biomass exposure were present in 30.9% of cases. We classified the pulmonary aspergillosis cases into aspergilloma (*n* = 12), chronic pulmonary aspergillosis (*n* = 431), and fungal pneumonia (*n* = 63) based on the radiological findings.

**Table 1:** Predisposing risk factors among the patients having suspected pulmonary aspergillosis in the present study.

Risk‐factor	Number of the sample (*n*)	% of total sample
Anti‐tubercular treatment	1389	34.33
SARS‐CoV‐2 infection	890	21.99
Lung parenchymal disorder	1389	34.33
Neutropenia	607	15.00
Malignancy	1397	34.52
Immune suppression	405	10.00
Diabetes mellitus	1741	43.03
Addiction and biomass exposure	1250	30.89

### 3.2 Identification of *Aspergillus* Species Causing LRTI

**Figure 2:**
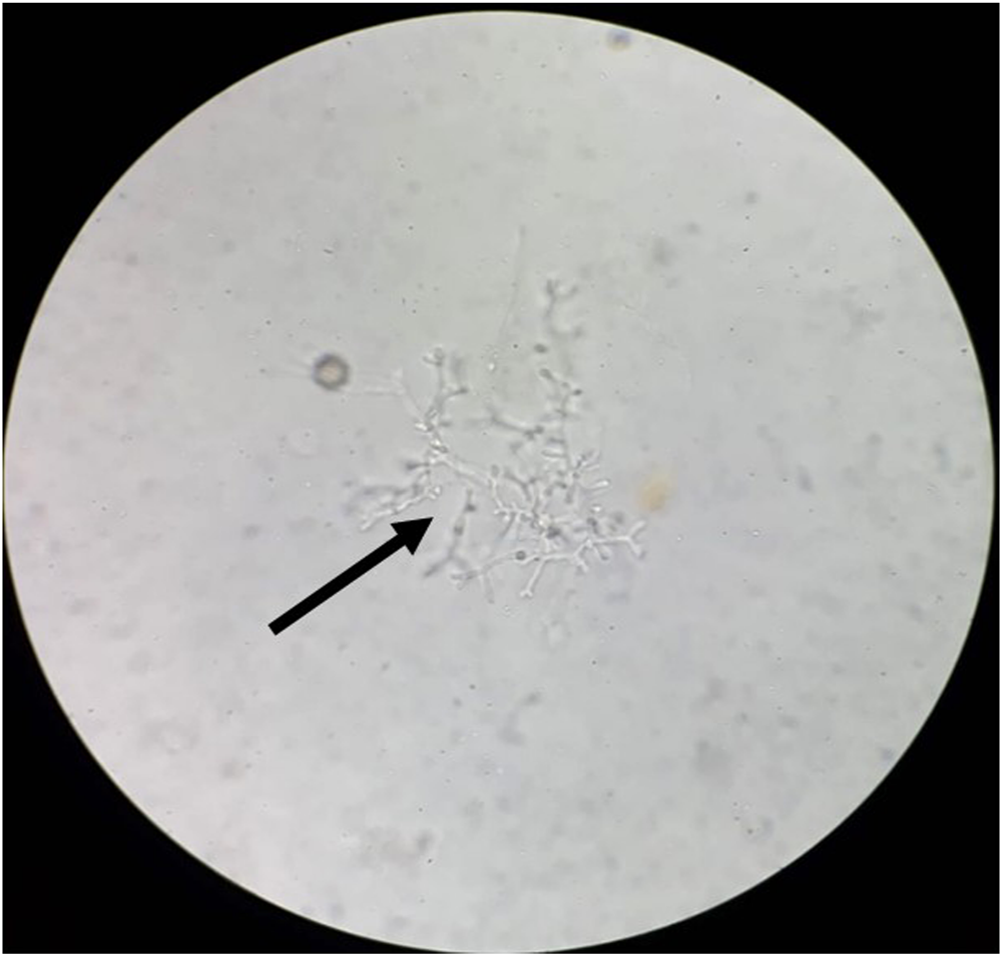
Septate branched hyaline hyphae of *Aspergillus* sp. in KOH wet mount of a bronchoalveolar lavage sample.

**Figure 3:**
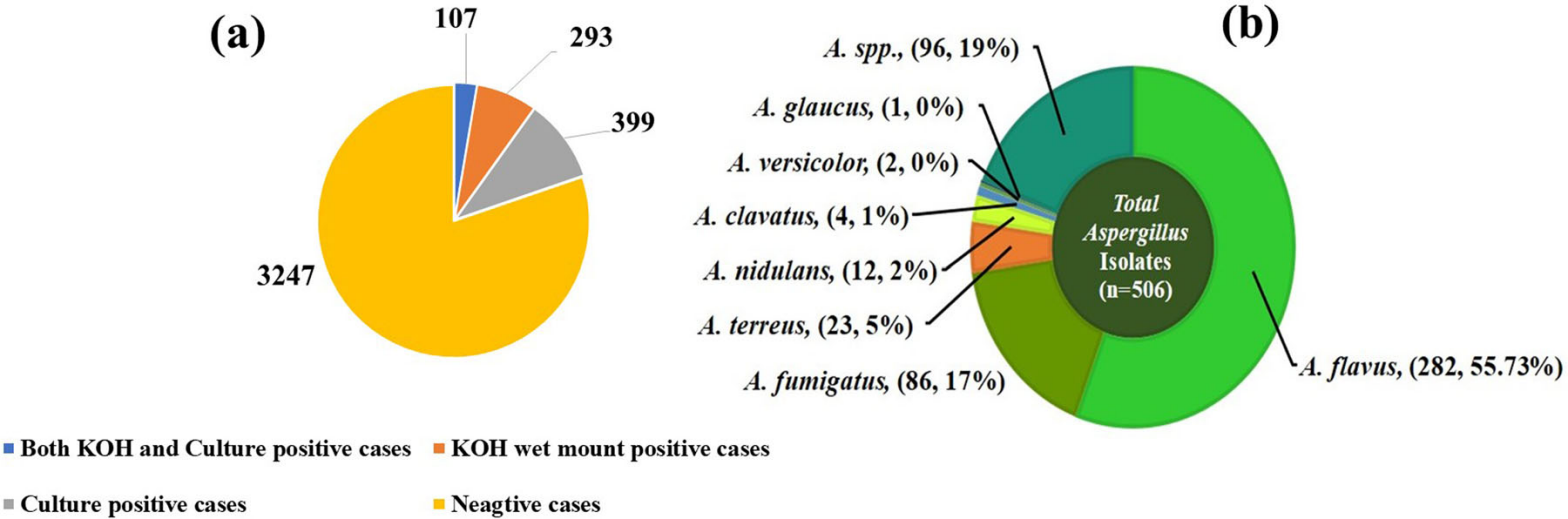
(a) Direct microscopy and culture‐based diagnosis of respiratory aspergillosis samples and (b) spectrum of *Aspergillus* species causing respiratory aspergillosis.

**Figure 4:**
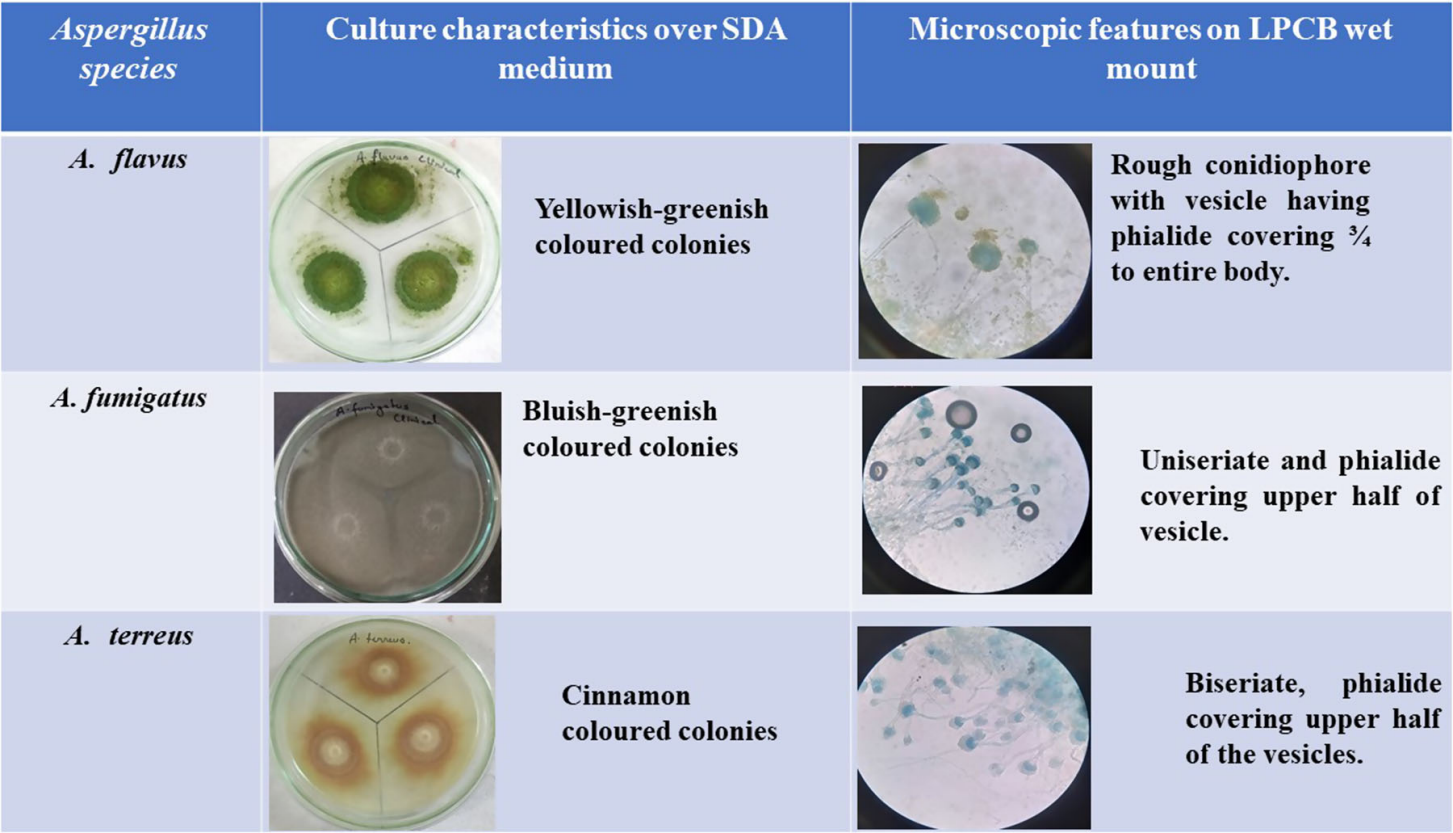
Phenotypic characteristics of isolated *Aspergillus* species causing respiratory aspergillosis.

**Figure 5:**
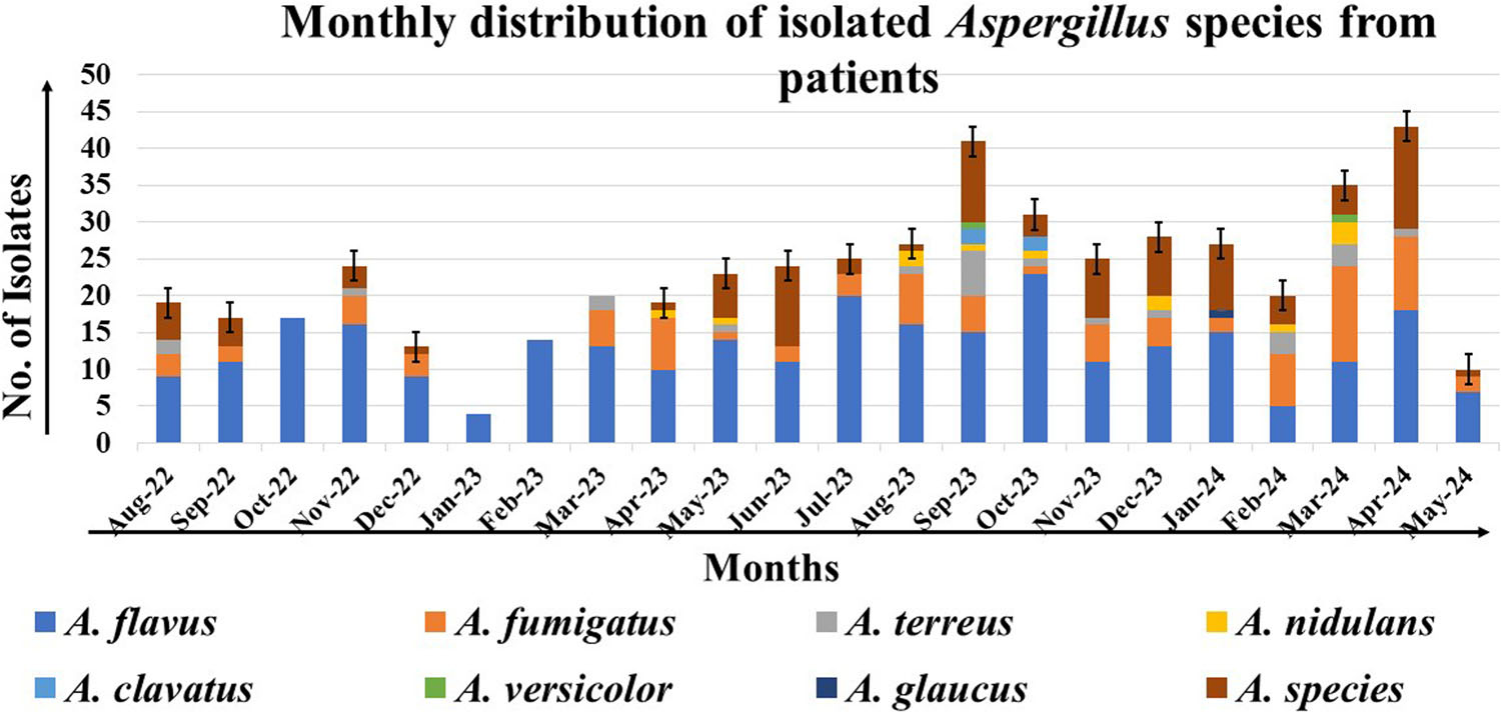
Time span of different *Aspergillus* species isolated from the respiratory samples of eastern Indian patients.

We processed all samples in accordance with standard mycological procedures. In 7.2% of the respiratory samples, we identified septate hyaline hyphae with acute angle branching, as depicted in Figure 2. Furthermore, we isolated 506 *Aspergillus* strains from these samples. Among them, 2.6% were positive for both KOH and culture isolates (Figure 3a). The variety of *Aspergillus* species isolated is shown in Figure 3b. The *A. flavus* complex emerged as the most frequently isolated species, accounting for 282 cases (55.7%), followed by the *A. fumigatus* complex, which was present in 86 samples (17%). Additionally, we identified *A. terreus* (23/506, 4.5%), *A. nidulans* (12/506, 2.4%), *A. clavatus* (4/506, 0.8%), *A. versicolor* (2/506, 0.4%), and *A. glaucus* (1/506, 0.2%) as contributors to human respiratory infections (Figure 3b). Aspergillus species were identified using phenotypic methods, focusing on culture characteristics and microscopic features as shown in Figure 4. However, 96 isolates (19%) could only be identified at the genus level and were categorized as unidentified *Aspergillus* species, emphasizing the need for advanced techniques such as MALDI‐TOF for precise species identification. We also observed seasonal variations in the *Aspergillus* species isolated from human infections, with *A. flavus* being the most prevalent, followed by *A. fumigatus*. Additionally, a higher number of *Aspergillus* species were isolated in September 2023 and April 2024 (Figure 5).

### 3.3 Antifungal Susceptibility Profile of Isolated *Aspergillus* Species

We assessed the antifungal susceptibility of the six antifungal agents against the isolated *Aspergillus* species using the broth microdilution technique, and the findings are presented in Tables 2 and 3. The MIC range observed for AMB was 0.03–16 µg/mL, for RUV it was 0.031–8 µg/mL, and for ITZ, VOR, and PCZ, it was 0.031–4 µg/mL across all *Aspergillus isolates* responsible for respiratory tract infections. *A. flavus* strains (*n* = 282), the MIC_50_ and MIC_90_ values were 2,4; 0.5, 1; 0.5, 2; 0.25, 0.5; and 1,2 for AMB, ITZ, VOR, PCZ, and RUV, respectively, whereas for CAS, both MIC_50_ and MIC_90_ were 0.062 µg/mL. Additionally, 91.8%, 93.6%, 97.5%, 98.6%, and 99.6% of the isolated strains were wild‐type for AMB, ITZ, VOR, POS, and CAS, respectively. *A. fumigatus* strains (*n* = 86), the MIC_50_ and MIC_90_ were 0.5,2; 0.5, 1; 0.5,1; 0.125, 2; and 1,2 for AMB, ITZ, VOR, PCZ, and RUV, respectively, with CAS having both MIC_50_ and MIC_90_ at 0.062 µg/mL. Furthermore, 90.7%, 95%, 94.2%, and 100% of *A. fumigatus* strains were wild‐type for AMB, ITZ, VOR, and CAS, respectively. Similarly, *A. terreus* strains (*n* = 23), the MIC_50_ and MIC_90_ were 4,8; 1,2; 1,2; 0.5,1; 1,2, and 0.062, 0.25 for AMB, ITZ, VOR, PCZ, RUV, and CAS; in contrast, 73.9% and 86.9% of *A. terreus* strains were wild‐type for AMP and CAS, respectively. However, all 23 strains were wild‐type for ITZ, VOR, and POS.

**Table 2:** Antifungal susceptibility profile of common *Aspergillus* species isolated from respiratory samples.

*Aspergillus* species	Antifungal drugs	MIC/MEC ranges	MIC/MEC_50_	MIC/MEC_90_	Interquartile (Q3‐Q1)	Geometric mean	ECV	WT (*n*, %)	NWT (*n*, %)
*A. flavus* (*n* = 282)	AMB	0.25–16	2	4	2	2.137	4	259 (91.84%)	23 (8.15%)
	ITZ	0.031–4	0.5	1	0.937	0.279	1	264 (93.61%)	18 (6.38%)
	VOR	0.031–4	0.5	2	1.25	0.583	2	275 (97.51%)	7 (2.48%)
	PCZ	0.031–1	0.25	0.5	0.437	0.182	0.5	278 (98.58%)	4 (1.41%)
	RUV	0.031–4	1	2	1.5	1.103	ND	ND	ND
	CAS	0.031–1	0.062	0.062	0.031	0.048	0.5	281 (99.64%)	1 (0.35%)
*A. fumigatus* (*n* = 86)	AMB	0.03–4	0.5	2	0.875	0.339	2	78 (90.69%)	8 (9.30%)
	ITZ	0.03–2	0.5	1	0.875	0.313	1	82 (95.34%)	4 (4.65%)
	VOR	0.03–2	0.5	1	0.937	0.245	1	81 (94.18%)	5 (5.81%)
	PCZ	0.031–1	0.125	2	0.187	0.162	0.5	83 (96.51%)	3 (3.48%)
	RUV	0.0625–8	1	2	0.5	0.679	ND	ND	ND
	CAS	0.31–0.5	0.0625	0.0625	0.031	0.053	0.5	86 (100%)	0 (0%)
*A. terreus* (n = 23)	AMB	4–8	4	8	4	3.766	4	17 (73.91%)	6 (26.08%)
	ITZ	0.125–2	1	2	1.5	0.739	2	23 (100%)	0
	VOR	0.125–2	1	2	0.5	0.675	2	23 (100%)	0
	PCZ	0.031–1	0.5	1	0.437	0.228	1	23 (100%)	0
	RUV	0.0625–4	1	2	1.5	0.86	ND	ND	ND
	CAS	0.031–0.25	0.0625	0.25	0.094	0.07	0.12	20 (86.95%)	3 (13.04%)

Abbreviations: ECV, epidemiological cut‐off value; MIC, minimum inhibitory concentration; MEC, minimum effective concentration; NWT, non‐wild type; ND, not determined; WT, wild type. (amphotericin B (AMB), itraconazole (ITZ), posaconazole (PCZ), ravuconazole (RUV), caspofungin (CAS), and voriconazole (VOR)).

**Table 3:** MIC and MEC ranges for less common causative *Aspergillus* species isolated from respiratory samples.

	Antifungal drugs (µg/mL)					
*Aspergillus* isolates	AMB (MIC)	ITZ MIC	VOR MIC	PCZ MIC	RUV MIC	CAS MEC range
*A. nidulans* (*n* = 12)	0.125–4	0.031–2	0.062–1	0.031–0.25	0.031–1	0.031–0.25
*A. clavatus* (*n* = 4)	1–4	0.062–1	0.5–2	0.06–4	0.031–8	0.031–0.25
*A. versicolor* (*n* = 2)	1–2	0.125–0.5	0.5–1	0.031–0.062	0.062–0.125	0.062–0.125
*A. glaucus* (*n* = 1)	4	0.5	0.062	1	0.125	0.125

Abbreviations: MIC, minimum inhibitory concentration; MEC, minimum effective concentration. (amphotericin B (AMB), itraconazole (ITZ), posaconazole (PCZ), ravuconazole (RUV), caspofungin (CAS), and voriconazole (VOR)).

### 3.4 Statistical Analysis

**Figure 6:**
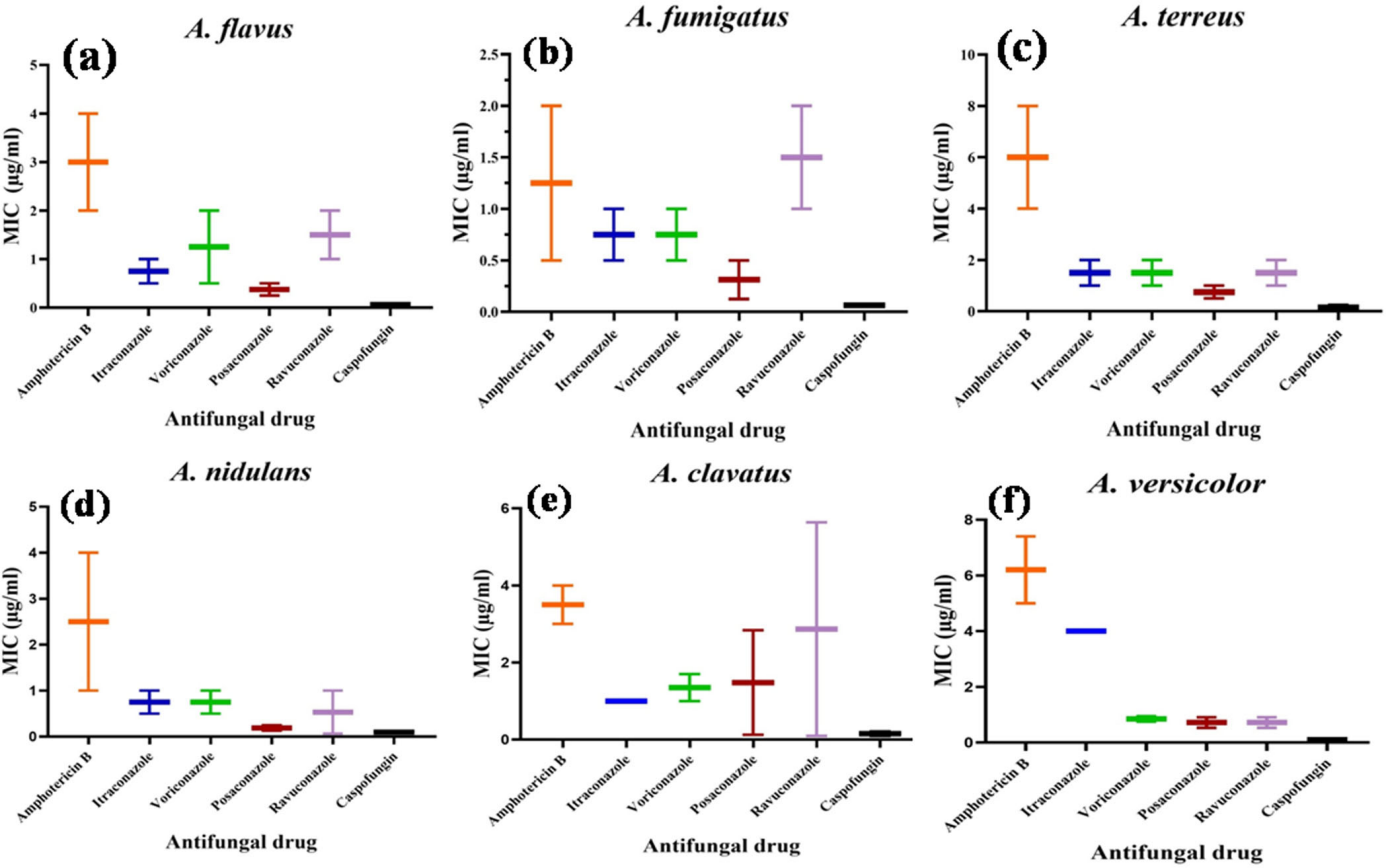
Whisker plot showing the MIC ranges of six antifungal drugs (Amphotericin B, itraconazole, posaconazole, ravuconazole, caspofungin, and voriconazole) against isolated *Aspergillus* species (a) *A. flavus,* (b) *A. fumigatus,* (c) *A. terreus,* (d) *A. nidulans,* (e) *A. clavatus,* (f) *A. versicolor*.

Tukey's multiple comparisons test and two‐way ANOVA were used in the statistical analysis to assess the variations in MIC_50_ and MIC_90_ values across several antifungal drugs (Figure 6). Higher drug concentrations were required to inhibit 90% of the isolates, and the MIC_90_ values were significantly higher than the MIC_50_ values. Significant variances with *p‐*value < 0.05 were also noted in the MIC_50_ and MIC_90_ values of several antifungal drugs, with AMB, PCZ, and CAS activity against *A. flavus* and *A. terreus;* in *A. versicolor*, significant MIC_50_ and MIC_90_ *p‐*values were noted against AMB, VOR, PCZ, RUV, and CAS. However, no significant *p‐*values were observed for *A. fumigatus*, *A. nidulans*, and *A. clavatus*.

## 4 Discussion

In developing countries, where pulmonary tuberculosis is prevalent, clinicians are concerned when patients exhibit LRTI with symptoms such as fever, cough, and hemoptysis. When pulmonary tuberculosis is suspected but not confirmed, pulmonary aspergillosis accounts for approximately 10% of these cases. The clinical symptoms of these diseases are often indistinguishable. In India, chronic pulmonary aspergillosis is a significant cause of morbidity and mortality (Denning et al. 2023). Males are more frequently affected than females, possibly due to outdoor activities, smoking, and pre‐existing lung parenchymal disorders, as shown in Figure 1 (Naj et al. 2024). Our study found a higher prevalence among male patients, although females were also affected when pre‐existing pulmonary tuberculosis and biomass exposure were risk factors (Table 1). We identified bronchiectasis, chronic obstructive pulmonary disease, and cavitary lung diseases (previous TB) as major predisposing lung deformities that facilitate fungal colonization and symptoms (Maduakor et al. 2020; Bobba and Arsura 2004). Uncontrolled hyperglycemia has been linked to aspergillosis (Liu et al. 2025). Elevated glucose levels promote *Aspergillus* conidia germination and lead to functional neutropenia by releasing immature neutrophils. Neutrophils are crucial for defense against *Aspergillus* species This increase in glucose levels occurs in patients taking steroids for autoimmune disorders. A meta‐analysis reported a higher risk of invasive aspergillosis in patients with diabetes (Liu et al. 2025). Additionally, a 19.8% prevalence of diabetes among Indians aged 45 and older has been reported, with similar rates in men and women (Sekher et al. 2025). Previous viral infections, such as SARS‐CoV‐2 and influenza, have been associated with *Aspergillus* tracheobronchitis and invasive pulmonary aspergillosis. These infections cause endothelitis and immune dysregulation, thereby increasing the risk of fungal infection. Recently, SARS‐CoV‐2 infection has been linked to pulmonary mucormycosis and aspergillosis (Chakravarty et al. 2022). Inappropriate steroid and zinc supplementation predispose individuals to invasive fungal infections (Nikhil et al. 2024; Denning et al. 2023). Respiratory aspergillosis typically occurs during the dry season, notably in April, September, October, and November, when fungal spores remain airborne, with lower detection in January, February, and March (Nageen et al. 2023). This seasonal variation may be due to patient selection from hospitals in this study (Figure 5).

In evidence‐based medicine, an accurate diagnosis of pulmonary aspergillosis requires the identification of characteristic hyphae, cultures, or serological tests. The most common method for detecting hyphae in respiratory samples is KOH wet mount (Figures 2 and 4). Although this method is specific, its sensitivity is low, allowing for antifungal treatments when clinical and radiological evidence suggests pulmonary aspergillosis. Fungal cultures are time‐consuming and labor‐intensive, with low isolation rates and high risk of contamination. However, we identified characteristic hyphae in 7.2% of respiratory samples. Respiratory aspergillosis can manifest as allergic reactions, aspergilloma, chronic, and invasive pulmonary aspergillosis, affecting various organs, particularly the brain (Gupta 2024). Aspergilloma and chronic pulmonary aspergillosis are typically caused by *A. flavus*, whereas invasive pulmonary aspergillosis is often caused by *A. fumigatus*. Other species, such as *A. niger, A. terreus, A. nidulans, A. glaucus*, and *A. versicolor*, also cause respiratory aspergillosis (Lass‐Florl et al. 2021). In our study, *A. flavus*, followed by *A. fumigatus*, was the primary cause of pulmonary aspergillosis in the enrolled patients (Figure 3). Rudramurty et al. reported similar findings regarding the causative agents of respiratory aspergillosis in the Indian population (Rudramurthy et al. 2019). However, a multicenter study by Ragozzino et al. found *A. fumigatus* to be predominant in respiratory samples (Ragozzino et al. 2022). This variation may be attributed to the endemicity of pulmonary tuberculosis in the Indian population, which leads to lung parenchymal deformities. Inhaled *A. flavus* conidia may colonize deformed respiratory bronchi and lung parenchyma, resulting in chronic pulmonary aspergillosis (Lakhtakia et al. 2022; Cioboata et al. 2025). In countries with low endemicity, small *A. fumigatus* conidia can lead to invasive pulmonary aspergillosis (O'Gorman 2011). The emergence of respiratory infections caused by *A. terreus, A. glaucus*, and *A. nidulans* as shown in (Figure 3b) has been documented in Spain, Austria, and Israel (Rattner and Sutherland 2016).

The management of respiratory aspergillosis necessitates the use of antifungal medications at the correct dosage while addressing the underlying risk factors. Triazoles, including itraconazole, voriconazole, posaconazole, and ravuconazole, are fungicidal agents that target ergosterol biosynthesis, a crucial component of fungal cell membranes. These drugs are the primary treatments for pulmonary aspergillosis. Although most *Aspergillus* spp. are susceptible to triazoles, resistance remains a significant challenge (Cadranel et al. 2012; Dladla et al. 2024). Polyenes, such as amphotericin B, create pores in fungal cells, whereas echinocandins inhibit β‐glucan synthesis. We evaluated the antifungal MIC against *Aspergillus* strains causing human infections and classified them into wild‐type and NWT strains based on CLSI guidelines. Among the azoles, itraconazole and voriconazole are the most frequently utilized ones to treat respiratory aspergillosis. Posaconazole is used as a prophylactic and adjunct therapy for aspergillosis and mucormycosis, whereas ravuconazole is a newer triazole antifungal. We observed higher MIC_50_ and MIC_90_ values for ravuconazole and lower MIC values for posaconazole. However, posaconazole is expensive and often unavailable in India. NWT strains of *A. flavus* and *A. fumigatus* show increased resistance to voriconazole and itraconazole, with a higher prevalence of itraconazole resistance. These findings align with those of Rosso F et al. who reported 8% and 6% resistance to ITZ and VOR, respectively, in isolated *Aspergillus* species (Rosso 2021). Interestingly, no non‐wild *A. terreus* strain was detected for the tested azoles. Moglad et al. found 100%, 44.4%, and 54.5% itraconazole resistance against isolated *A. niger, A. fumigatus*, and *A. flavus* strains, respectively, while voriconazole showed high efficacy against *Aspergillus* species in patients with lung disorders (Moglad et al. 2022). In a multicenter study, Badiee et al. identified NWT *Aspergillus* species for AMB (3%), VOR (1.3%), PCZ (2.6%), isavuconazole (1.7%), and CAS (4.7%) from various samples (Badiee et al. 2022). We observed slightly higher NWT *Aspergillus* strains for the tested antifungal agents. Pfaller et al. reported varying posaconazole susceptibility against *A. fumigatus* regionally, with 2.1%, 2.2%, 1.8%, and 0.7% non‐wild strains in Europe, North America, Latin America, and Asia‐Pacific, respectively. We found 1.41% non‐wild *A. flavus* strains for posaconazole, possibly due to its limited availability and high cost in Southeast Asia (Pfaller et al. 2021).

Liposomal AMB is the preferred treatment for azole‐resistant respiratory *Aspergillus* infections. We observed higher MIC_50_ and MIC_90_ values for *A. terreus* and *A. flavus* in the respiratory samples. Our findings indicated that 26%, 8%, and 4% of the non‐wild strains of *A. terreus, A. flavus*, and *A. fumigatus*, respectively, were resistant to AMB (Tables 2 and 3). Previous studies have reported an increase in the number of AMB‐resistant strains. Fakhim et al. documented AMB resistance rates of 36.8%, 14.9%, 5.2%, and 2.01% in *A. terreus, A. flavus, A. niger*, and *A. fumigatus*, respectively (Fakhim et al. 2022). A surveillance study at a tertiary care hospital revealed that 67.2% of *Aspergillus* isolates had elevated AMB MIC, potentially leading to treatment failure (da Fonseca et al. 2023). This resistance may be attributed to the frequent use of AMB in treating invasive candidiasis, mucormycosis, cryptococcosis, and disseminated histoplasmosis (Cavassin et al. 2021). Echinocandins, such as CAS, anidulafungin, and micafungin, inhibit β‐glucan synthesis, which is a crucial component of fungal cell walls. As fungistatic agents, they compromise cell wall integrity and are used as salvage therapy for pulmonary aspergillosis. In this study, 13.04% of *A. terreus* isolates were NWT for caspofungin, whereas 100% and 99% of *A. fumigatus* and *A. flavus* isolates were wild‐type, respectively (Tables 2 and 3). Kaur et al. reported 100% CAS susceptibility in all *Aspergillus* isolates (Kaur et al. 2024). Yassin et al. noted MIC_50_ and MIC_90_ values of 0.063 and 1 μg/mL, respectively, for CAS against *Aspergillus* species, although they identified a single resistant *A. flavus* isolate (Yassin et al. 2021). This lower resistance may be due to its limited use in developing countries.

*A. flavus, A. fumigatus*, and *A. terreus* are the primary fungi responsible for pulmonary aspergillosis, although species such as *A. nidulans, A. glaucus*, and *A. versicolor* have also been implicated (Kousha et al. 2011). Although these fungi generally respond to antifungal treatments, resistance remains a significant concern. Notably, *A. nidulans* strains exhibit higher AMB MIC levels (Tavakoli et al. 2020). In our study, strains of *A. nidulans, A. glaucus*, and *A. clavatus* showed an AMB MIC of 4 μg/mL (Table 3). Araujo et al. reported 50 environmental and one clinical isolate of *A. glaucus*, all sensitive to antifungal agents with an MIC range of 0.03–2 µg/mL (Araujo et al. 2007). Similarly, Kaur et al. observed low MIC values for AMB, ITZ, VOR, and CAS against *A. glaucus* (Kaur et al. 2024). A rare case of fatal brain infection caused by *A. glaucus* was documented (Tavakoli et al. 2020). An *A. clavatus* isolate with MIC values of 4 and 8 μg/mL for AMB and RUV, respectively, was also noted. The diverse *Aspergillus* species causing infections and increasing drug resistance underscore the need for direct microscopy, culture isolation, and antifungal susceptibility testing in suspected cases of pulmonary aspergillosis, particularly in regions with high tuberculosis prevalence. This study addresses the gap in regional epidemiology and resistance patterns of *Aspergillus* species in Eastern India, detailing the prevalence of species in respiratory infections and their seasonal distributions. However, the limitations of this study include the absence of phenotypic characterization of unidentified *Aspergillus* species using molecular techniques and MALDI‐TOF. We did not employ serological tests to diagnose pulmonary aspergillosis or to assess the treatment response. Despite these limitations, this study will aid in managing pulmonary aspergillosis by identifying the causative species and determining antifungal MIC. Identifying resistance trends in specific areas contributes to the epidemiological mapping of fungal infections. This study provides baseline data for future research on resistance evolution, mutation tracking, and therapeutic innovations.

## 5 Conclusions

In countries where tuberculosis (TB) is widespread, such as India, patients who exhibit symptoms such as coughing with sputum are often treated with antitubercular medications, even when laboratory tests return negative results. In addition to TB, pulmonary aspergillosis presents with similar symptoms. Therefore, a high clinical suspicion and prompt diagnosis of pulmonary aspergillosis using KOH wet mounts can help address this issue. However, obtaining suitable samples, such as BAL fluid, poses a significant challenge in developing nations. In every suspected case, direct microscopic examination with a KOH wet mount should be conducted. Additionally, the interpretation of fungal cultures should always consider the clinical and radiological contexts in both immunocompetent and immunosuppressed patients. Although *Aspergillus* species generally respond well to antifungal treatments, drug resistance is a growing concern. Consequently, for each suspected case of pulmonary aspergillosis, both KOH wet mount and fungal cultures with antifungal susceptibility testing should be performed. This approach can not only decrease morbidity and mortality but also prevent the inappropriate use of antitubercular drugs. Furthermore, identifying resistance patterns in specific regions aids in the epidemiological mapping of fungal infections.

## Author Contributions

**Aishwarya Nikhil:** conceptualization, investigation, formal analysis. **Sradha Choudhury:** conceptualization, writing – original draft, investigation, formal analysis. **Mohit Bhatia:** writing – review and editing. **Atul Kumar Tiwari:** conceptualization, writing – original draft. **Ritika Srivastava:** writing – original draft, investigation, formal analysis. **Abhirami Prasad:** writing – original draft, investigation, formal analysis. **Ragini Tilak:** writing – review and editing. **Munesh K. Gupta:** supervision, writing – original draft, writing – review and editing. **Roger J. Narayan:** supervision.

## Conflicts of Interest

The authors declare no conflicts of interest.

## Data Availability

All the data are presented in the manuscript: any raw data can be available by request to the first and corresponding author (email: muneshg.micro@bhu.ac.in; aishwaryanikhil1995@gmail.com).
